# The Proximate Causes of Sexual Size Dimorphism in *Phrynocephalus przewalskii*

**DOI:** 10.1371/journal.pone.0085963

**Published:** 2014-01-21

**Authors:** Wei Zhao, Nai-fa Liu

**Affiliations:** School of Life Sciences, Lanzhou University, Lanzhou, China; Clemson University, United States of America

## Abstract

Sexual size dimorphism (SSD) is a common phenomenon and is a central topic in evolutionary biology. Recently, the importance of pursuing an ontogenetic perspective of SSD has been emphasized, to elucidate the proximate physiological mechanisms leading to its evolution. However, such research has seldom focused on the critical periods when males and females diverge. Using mark-recapture data, we investigated the development of SSD, sex-specific survivorship, and growth rates in *Phrynocephalus przewalskii* (Agamidae). We demonstrated that both male and female lizards are reproductively mature at age 10–11 months (including 5 months hibernation). Male-biased SSD in snout-vent length (SVL) was only found in adults and was fully expressed at age 11 months (June of the first full season of activity), just after sexual maturation. However, male-biased SSD in tail length (TL), hind-limb length (LL), and head width (HW) were fully expressed at age 9–10 months, just before sexual maturation. Analysis of age-specific linear growth rates identified sexually dimorphic growth during the fifth growth month (age 10–11 months) as the proximate cause of SSD in SVL. The males experienced higher mortality than females in the first 2 years and only survived better than females after SSD was well developed. This suggests that the critical period of divergence in the sizes of male and female *P. przewalskii* occurs between 10 and 11 months of age (May to June during the first full season of activity), and that the sexual difference in growth during this period is the proximate cause. However, the sexual difference in survivorship cannot explain the male-biased SSD in SVL. Our results indicate that performance-related characteristics, such as TL, HW, and LL diverged earlier than SVL. The physiological mechanisms underlying the different growth patterns of males and females may reflect different energy allocations associated with their different reproductive statuses.

## Introduction

Sexual dimorphism is a common and long-discussed feature of the animal world that is reflected in a variety of biological characteristics. Among these, sexual size dimorphism (SSD) is a widespread phenomenon observed in many taxa that is believed to have evolved through several non-exclusive ultimate selection pressures, including sexual selection, fecundity selection, and intersexual niche divergence [1]. However, these adaptive hypotheses explain relatively small proportions of the interspecific variance in lizard SSD [2] and relatively little is known about the proximate developmental mechanisms of SSD. Furthermore, the relative importance of ultimate versus proximate control of SSD is rarely known. Recently, the importance of an ontogenetic perspective, in which SSD is viewed as a developmental process of ‘growing apart’, has been emphasized [3] and the proximate causes of SSD has been determined in several lizards species under this framework [4]–[7].

For species that continue to grow after maturation, SSD can arise from multiple contributing factors, such as sexual differences in survivorship, age distribution, and growth trajectories [4], [7]–[9]. Sexual differences in age-specific growth have attracted much attention and are mainly caused by ecological differences between males and females, sex-specific trade-offs in allocation of energy between growth and reproduction, or sexual divergence in growth regulators. However, growth is strongly responsive to the feeding rate, and the development of SSD can be suppressed or eliminated under laboratory conditions [4]–[5]. Although it is clear that sex-specific growth plays an important role in SSD development, we still know little about when the divergence take place and whether the differences persist throughout life. There is also little information concerning the proximate physiological mechanisms involved in this differentiation and the factors that influence the process. To resolve these questions, we must first determine the critical periods when male and female sizes diverge.

Toad-headed lizards of the genus *Phrynocephalus* (Agamidae) include more than 40 species distributed from north-western China to Turkey and they are major components of the central Asian desert fauna. *P. przewalskii*, is widely distributed in northern China and adjacent Mongolia, and has been subjected to extensive systematic and ecological studies [10]–[17]. Juveniles achieve sexual maturity within 1 year from birth with males and females reaching snout-vent lengths (SVL) of 43 mm and 45 mm, respectively [10], [12]. Wang and Wang [11] indicated that this species exhibits a male-biased sexual dimorphism in body size and relative hind-limb length (LL), and they considered that this dimorphism arises by sexual selection. However the proximate causes of this SSD are unknown. Using mark-recapture methods, we analyzed ontogenetic changes in SSD, and in sex-specific survivorship and growth rates of *P. przewalskii*, and detected the critical periods during which male and female sizes diverge. This is important for our understanding of the proximate mechanisms and ultimate evolutionary pressures leading to sexual dimorphism in this species.

## Materials and Methods

### Study area and data collection

The study site, Alax Zuoqi (38.85°N, 105.63°E; 1448 m above sea level), Inner Mongolia, China is located at the northeastern margin of the Tengger Desert. The predominant land form is semi-desert grassland with a vegetation cover (mainly grass) of less than 15% [16]. The climate is semi-arid with a mean annual precipitation of 207.5±9.6 mm (n = 30 years) and mean annual temperature of 8.7±0.1°C (n = 30 years). A single mark-recapture quadrat of 50 m×50 m with a 20 m buffer zone was established in April 2008.

The quadrat was visited monthly between April and October (during the first third of the month), from 2008 to 2010. Lizards were collected by hand and numbered with a unique toe-clip, and their sex was determined by the presence or absence of hemipenes. Because it was difficult to distinguish male and female lizards until the hemipenes were well developed, the sex of juveniles was not determined until they were 3 months old. The following measurements were made for each lizard: SVL; tail length (TL); head width (HW); and LL (from the carpus to the elbow). Lizards that were first captured as hatchlings or early yearlings could be accurately aged (in months) and were used in the following analysis. Animals were released at the site of capture after measurements were taken.

### Sexual size dimorphism

All lizards for which gender and age could be determined were used in the SSD analysis. For each age class, the overall body size and other proportional measurements were compared between the two sexes. Analysis of variance (ANOVA) was used to examine the SVL difference between male and female lizards of the same age category, while analysis of covariance (ANCOVA) (using SVL as a covariate) was used to examine sex differences in TL, LL, and HW. To determine whether the SSD pattern varied among age classes, ANOVA and ANCOVA (using SVL as a covariate) were used with age and sex as factors and other length characteristics as dependent variables.

### Growth rate

Male and female growth in SVL under natural conditions was compared using two approaches. First, for each age category, linear growth rates between two censuses were compared directly. Growth rate was estimated by subtracting initial SVL from final SVL and dividing by elapsed time. Then, the correlation between growth rate and the mean SVL at initial and final capture was examined for each age category. If there was significant correlation, ANCOVA (using mean SVL as a covariate, sex as a factor) was used to detect the difference in growth rate between sexes, otherwise ANOVA was used. Capture intervals less than 15 days and greater than 60 days were not considered.

In the second approach, various growth models were fitted and compared between male and female lizards. We first calculated the size-specific growth rate (SGR) as the growth rate divided by the mean SVL of initial and final capture [18]. We then fitted the SGR and SVL of the two sexes to logistic, Gompertz and von Bertalanffy growth models, as described in Johnston [7]. The logistic model produced the highest value of *R* ^2^ from a linear regression of the original data and was chosen as the best model (Table 1). SVL data from accurately aged (in months) lizards were fitted to the best model and the asymptotic length (A) and the characteristic growth rate (k) were estimated and directly compared between the sexes. The SVL of the smallest measured neonate was set as the size at age zero (25.98 mm for both sexes), and absolute time was converted to ‘growth months’ by subtracting the period of winter dormancy (late October to the following late March, about 5 months).

**Table 1:** Parameters of the differential forms of the logistic, Gompertz and Von Bertalanffy growth madels for male and female *Phrynocephalus przewalskii* at Alax Zuoqi, China during the period 2008–2010.

Modle	Equation	R^2^
Males (n = 160)		
Logistic	SGR = 0.007008−0.000122SVL	0.495
Gompertz	SGR = 0.021236−0.005203lnSVL	0.486
Von Bertalanffy	SGR = −0.003462+0.215727(1/SVL)	0.473
Females (n = 192)		
Logistic	SGR = 0.009048−0.000175SVL	0.421
Gompertz	SGR = 0.027373−0.006895lnSVL	0.413
Von Bertalanffy	SGR = −0.004802+0.264825(1/SVL)	0.403

Probabilities show the significance of the Pearson correlation coefficients for each equation. N, sample size; R^2^, coefficient of determination; SGR, specific growth rate; SVL, snout-went length.

### Survivorship

Survivorship was determined by a lizard's continued presence in sequential censuses. When a lizard was absent from a census and was never seen again it was treated as a mortality at the first absence. Short-range emigration was checked by searching for marked individuals in the buffer zone around the quadrat. The survivorships of eggs and the hatching rate were not investigated in this study. The primary sex ratio was assumed to be 1∶1. Time-specific life tables for each sex were established using the estimated age-specific survivorship (lx), mortality rate (qx), and life expectation (ex), following Sun [19].

### Statistical analyses

All data were tested for normality and homogeneity of variances and were ln-transformed when necessary to achieve the conditions for using parametric tests. Statistical tests were performed using SPSS 19.0 for Windows. Values are expressed as means ±SE and *P*<0.05 was considered as statistically significant.

### Ethics Statement

Our experimental procedures complied with the current laws on animal welfare and research in China and were specifically approved by the Animal Research Ethics Committee of Lanzhou University. The capturing and tagging of animals were authorized by China's Ministry of Education.

## Results

A total of 327 lizards were captured and measured 945 times in this study. Among 176 lizards that were captured more than once, 105 individuals (52 male and 53 female lizards) were successfully sexed and aged, and a total of 352 linear growth rates (GR) were calculated for these 105 individuals. Juveniles mainly hatched in late July and early August. The oldest individuals whose age could be accurately determined were aged 19 months for both sexes.

### Sexual size dimorphism

#### Overall body size (SVL)

**Figure 1:**
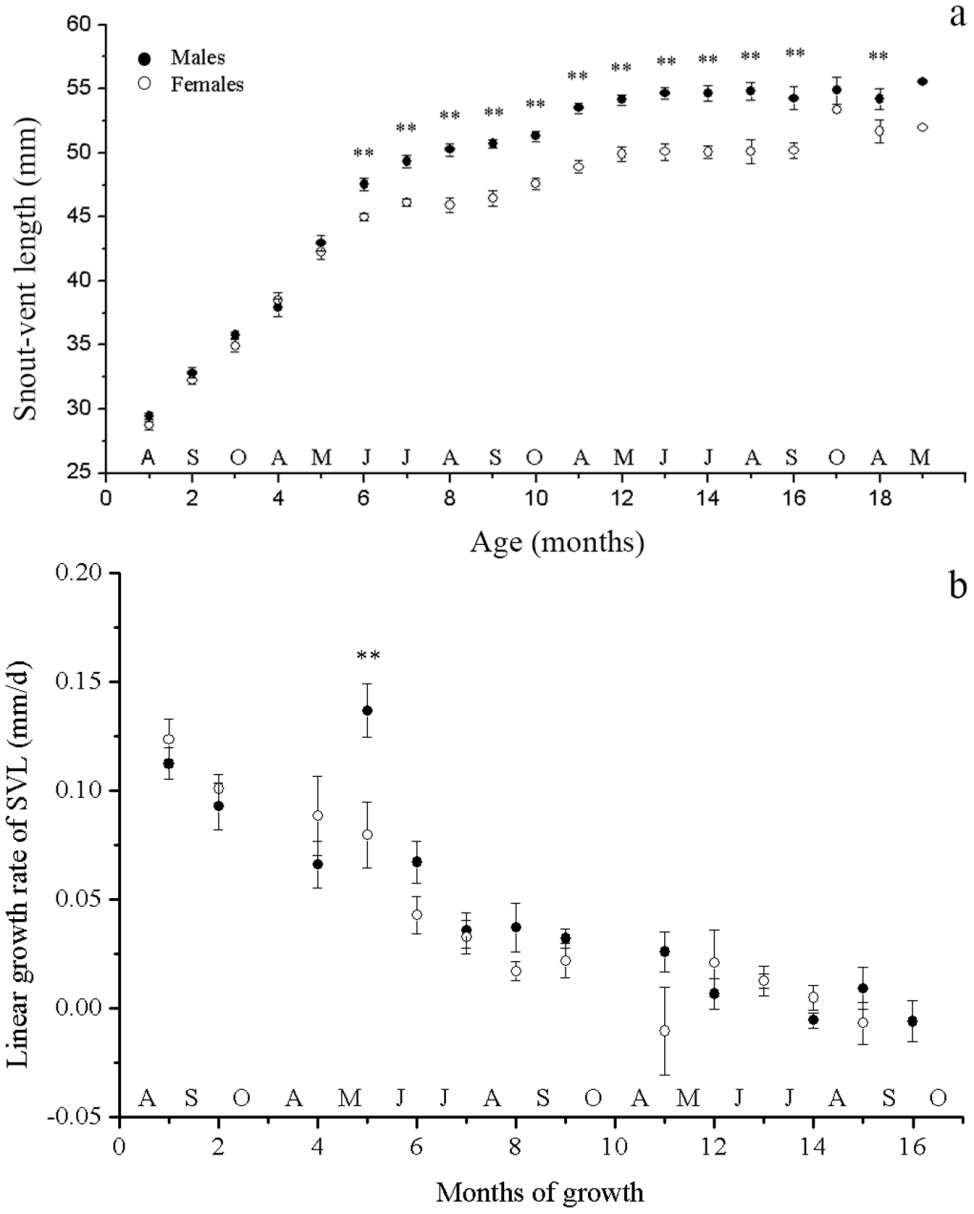
Size and linear growth rate of snout-vent length of male and female *Phrynocephalus przewalskii* in Alax Zuoqi, China. The data are presented as means ±SE. The black circles represent male lizards and the open circles represent female lizards. Data were collected using mark-recapture methods and the lizards were accurately aged from the month that they were first captured as hatchlings or early yearlings. Those larger than 2 years old when they first captured were eliminated. Individual lizards occur more than once in this figure and hatching was assumed to have occurred in August. Cohorts that hatched in different years are combined. Months represent the time spent growing (April–October), not actual ages. Lettering above the axis indicates the census date (month). Asterisks indicate significant size differences (a, ANOVA; b, ANCOVA with the mean SVL of initial and final capture as covariate; ** *P*<0.01; * *P*<0.05).

There was a strong age × sex interaction for overall body size (*F_18,507_* = 4.810, *P*<0.001). This suggested that the pattern of SSD in SVL exhibited ontogenetic variation. No significant sexual difference was found before 10 months old (May during the first full season of activity; Fig. 1a). This implies that female and male lizards do not differ in SVL before sexual maturity (see the following section). Males of 11 months old (June during the first season of full activity) were larger in SVL than females of the same age (*F_1,54_* = 2.312, *P*<0.001). Once this SSD pattern was established between the ages of 10 and 11 months (the following May and June after hatching), it was maintained for the remainder of the animals' lives (Fig. 1).

#### Proportional measurements

**Figure 2:**
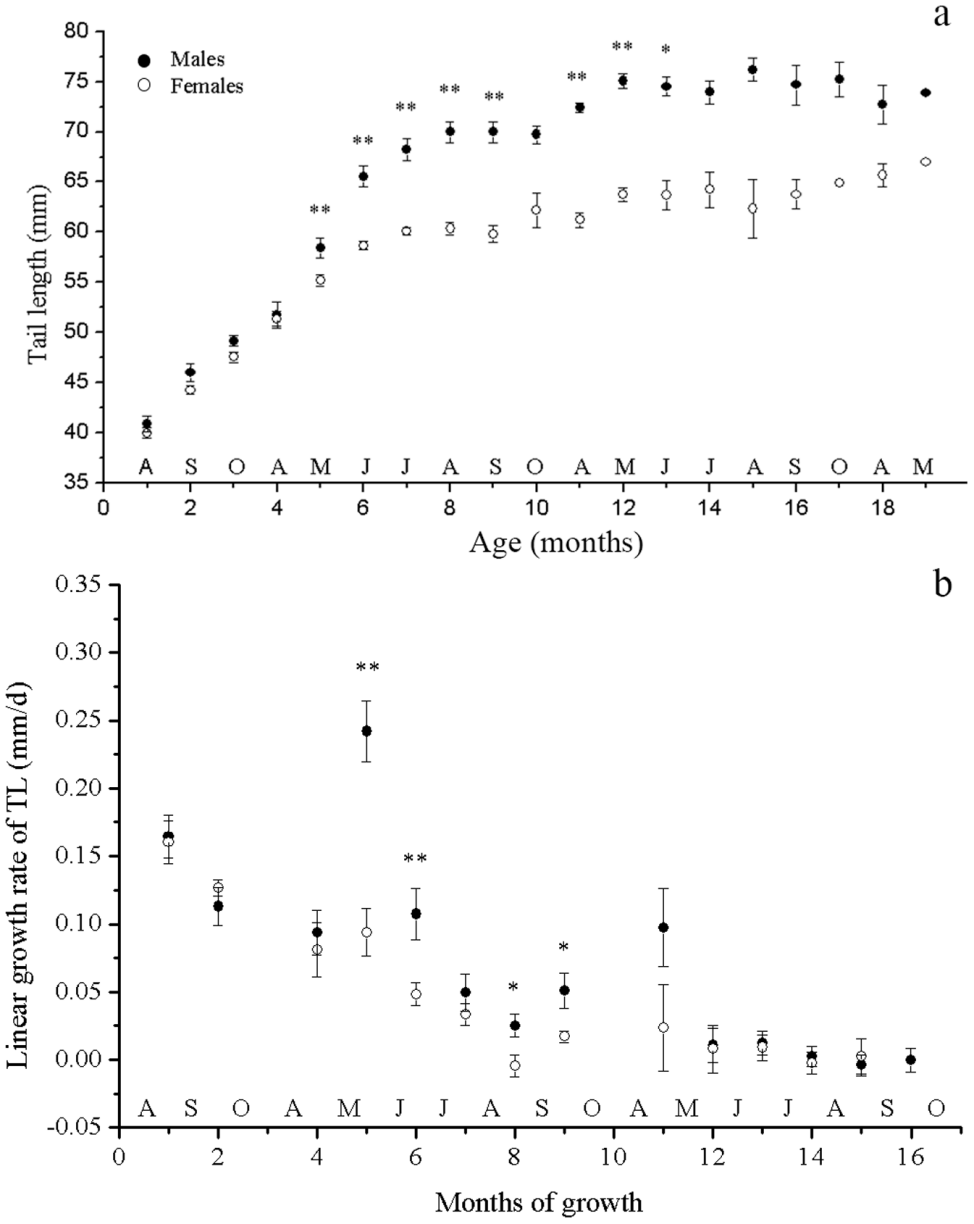
Size and linear growth rate of tail length of male and female *Phrynocephalus przewalskii* in Alax Zuoqi, China. The data are presented as means ±SE. The black circles represent male lizards and the open circles represent female lizards. Asterisks indicate significant size differences (a, ANCOVA with the SVL as covariate; b, ANOVA; ** *P*<0.01; * *P*<0.05).

**Figure 3:**
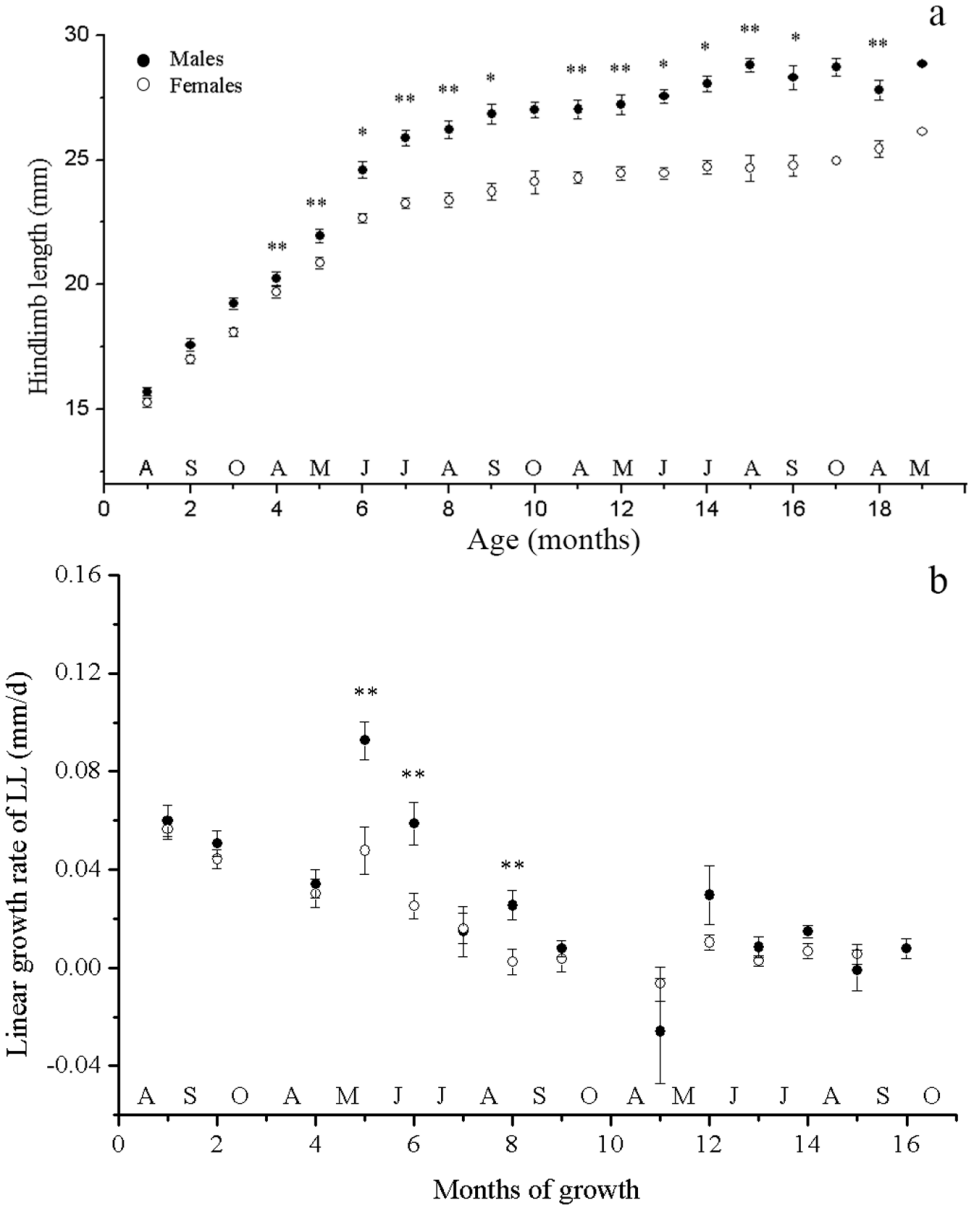
Size and linear growth rate of head width of male and female *Phrynocephalus przewalskii* in Alax Zuoqi, China. The data are presented as means ±SE. The black circles represent male lizards and the open circles represent female lizards. Asterisks indicate significant size differences (a, ANCOVA with the SVL as covariate; b, ANOVA; ** *P*<0.01; * *P*<0.05).

**Figure 4:**
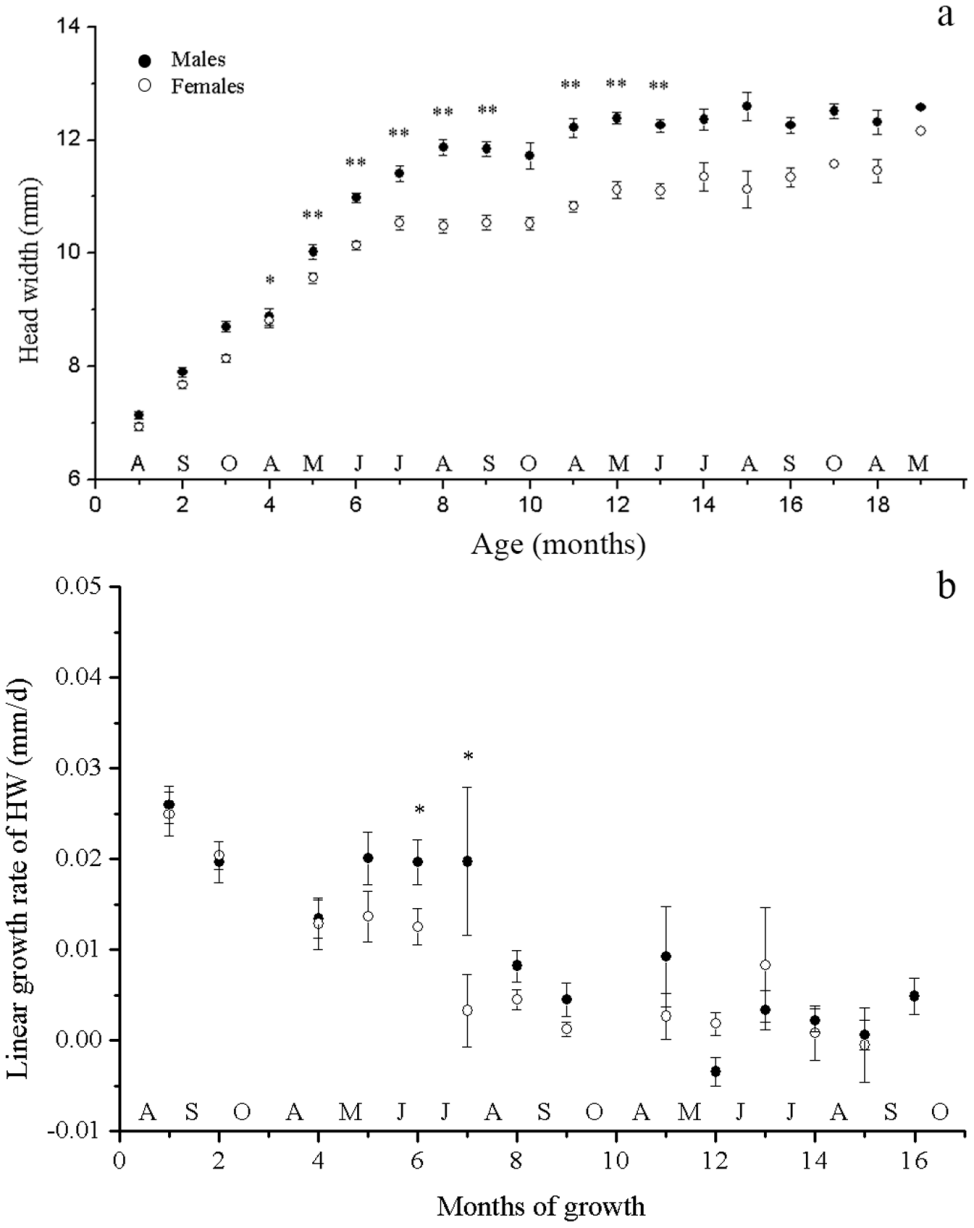
Size and linear growth rate of hindlimb length of male and female *Phrynocephalus przewalskii* in Alax Zuoqi, China. The data are presented as means ±SE. The black circles represent male lizards and the open circles represent female lizards. Asterisks indicate significant size differences (a, ANCOVA with the SVL as covariate; b, ANOVA; ** *P*<0.01; * *P*<0.05).

Interactions of age × sex were also found for the other proportional measurements (ANCOVA, *P*<0.005 in each case). Unlike SVL, the SSD of all the other characteristics were well established by the age of 10 months (May during the first season of full activity) with male lizards having relatively larger HW, TL, and LL (ANCOVA, *P*<0.010 in each case; Figs. 2–4). Once this SSD pattern was established at the age of 9–10 months (April and May following hatching), it was maintained for the remainder of their lives (Figs. 2–4).

In summary, patterns of SSD in SVL and proportional body measurements exhibited ontogenetic variation. Sexual dimorphism in SVL was not significant until sexual maturity but the SSD in other measured characteristics was fully expressed before sexual maturation. Once the SSD pattern was established it was maintained throughout further life. May and June following hatching were the critical periods in which male and female sizes diverged.

### Linear growth rate

#### Overall body size

A total of 352 linear growth rates (GR) were calculated for 105 individuals in this study. The growth rate of SVL decreased significantly as SVL increased, for both sexes (male: *r* = −0.571, *P*<0.001; female: *r* = −0.518, *P*<0.001), and the slope was similar between the two sexes (ANCOVA *F_1,348_* = 3.345, *P* = 0.064). Stronger age × sex interaction was found in the GR of SVL (*F_12,322_* = 2.444, *P* = 0.005); thus there was an ontogenetic variation of growth pattern. The main period of decrease of SVL growth rate took place at the age of 11–12 months (Fig. 1b). The growth rate of SVL in male lizards was significantly greater than in female lizards only in the fifth growing months (May to July during the first full season of activity, Fig. 1b). This difference occurred just before the establishment of SSD.

#### Proportional measurements

**Figure 5:**
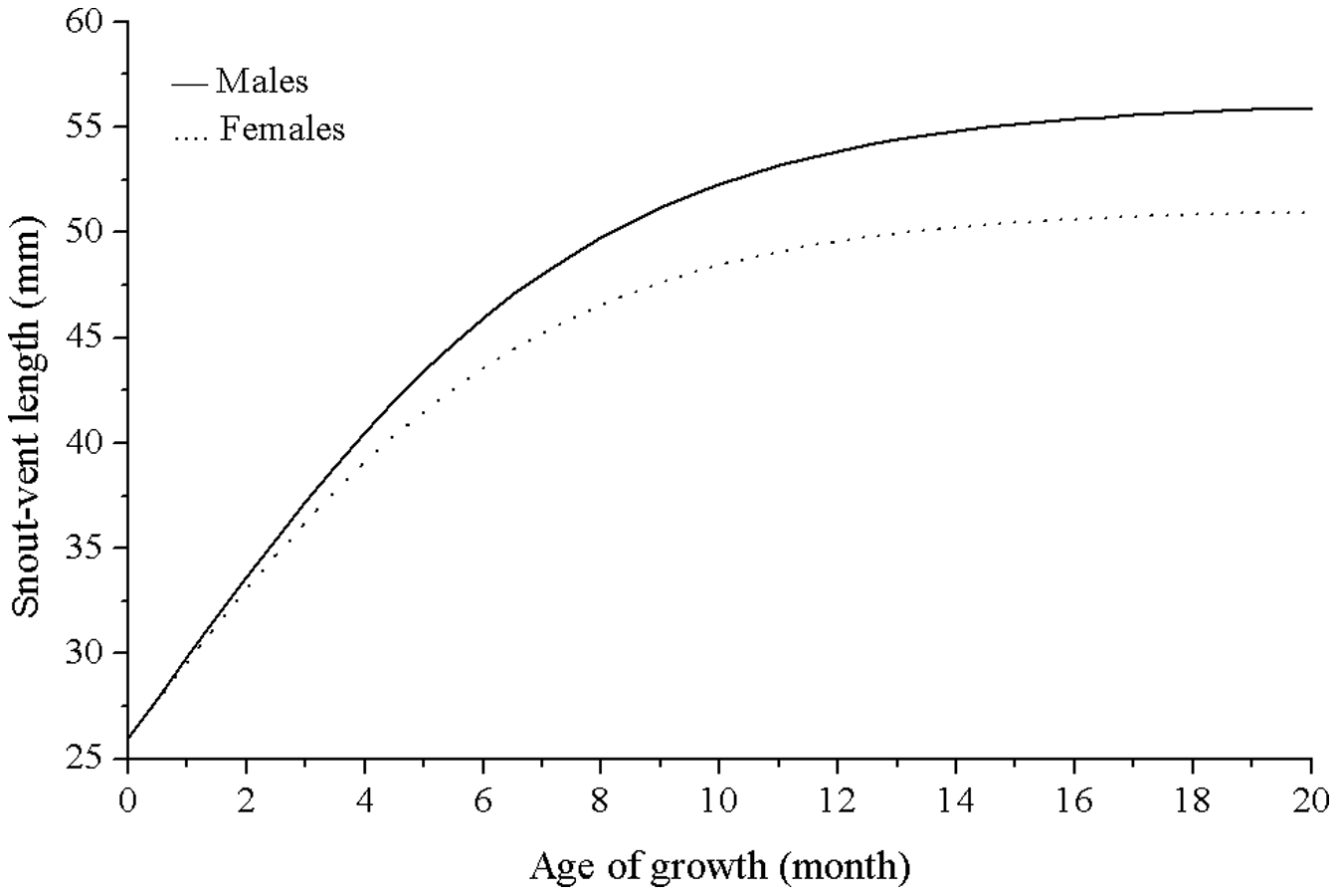
Logistic growth curves for snout-vent length of male and female *P. przewalskii* in Alax Zuoqi, China. Months represent time spent growing (actual age minus 5 months of dormancy). Individual lizards occur more than once in this figure and hatching is assumed to have occurred in August. The continuous line represents male lizards and the dotted line represent female lizards.

The growth rates of other characteristics also decreased significantly as SVL increased, for both male and female lizards (ANOVA *P*<0.01 in all cases). Age × sex interactions were found for the growth rates of TL and LL (*F_12,322_* = 4.336, *P*<0.001; *F_12,322_* = 2.456, *P* = 0.004, respectively) but not for HW (*F_12,322_* = 1.630, *P* = 0.082). For TL and LL, the difference between the growth rate of male and female lizards mainly occurred in the fourth, fifth and eighth growth months, with male lizards growing faster than female lizards (ANOVA *P*<0.028 in all cases; Figs. 3b, 4b). HW grew faster in male lizards than in female lizards during the sixth growth months (ANOVA *F_1,48_* = 5.106, *P* = 0.028; Fig. 5b).

### Growth model

The logistic model fitted our data best for both sexes and explained 49.5% of the variance in SGR in male lizards and 42.1% in female lizards (Table 1). The asymptotic size attained by male lizards was 56.17±0.69 mm (±95% CI) and was significantly larger than for female lizards (51.13±0.73 mm). By contrast, male lizards (0.275±0.012) and female lizards (0.286±0.016) exhibited similar characteristic growth parameters (Fig. 5).

Male lizards larger than 45 mm and female lizards larger than 43 mm SVL had reproductive potential and these were considered to be the smallest individuals exhibiting sexual maturity [12], [17]. According to our estimated growth curves, male lizards achieve sexual maturity at 10.6 months and female lizards at 10.7 months. Our mark-recapture data also confirmed that male lizards reached 45 mm at 10–11 months and female lizards reached 43 mm at 10–11 months. Asymptotic body size was reached by the end of the second full growth season (at about age 30 months).

### Survivorship

Survivorship and time-specific life tables for the two sexes were computed based on the age-specific mortality rates between July 2008 and June 2009 (Table 2). The pattern of age-specific survivor rates was similar between male and female lizards with yearlings and older individuals having relatively lower survivor probability than other ages (Table 2). However female lizards had relatively higher survivorship than male lizards of the same age. The mortality of male lizards was higher than that of female lizards during the first 3 years of age, but was lower in the last 2 years. The life expectancy for male lizards was lower than that of female lizards during the first 2 years. By the end of their third year, male lizards had a higher life expectancy than female lizards of the same age (Table 2).

**Table 2:** Time specific life tables for males and females *Phrynocephalus przewalskii* at Alax Zuoqi, China during the period 2008–2009.

Age	Males				Females			
x	n_x_	s_x_	l_x_	e_x_	n_x_	s_x_	l_x_	e_x_
0	1000	0.339	1.000	1.067	1000	0.339	1.000	1.206
1	339	0.414	0.339	1.173	339	0.476	0.339	1.583
2	140	0.250	0.140	1.125	161	0.750	0.161	1.775
3	35	1.000	0.035	2.000	121	0.500	0.121	1.200
4	35	0.500	0.035	1.000	61	0.400	0.061	0.900
5	18	0.000	0.018	0.500	24	0.000	0.024	0.500

n_x_, frequency of lizards living to age x; s_x_, age specific survival rate of age x; l_x_, the probability of survival from birth to age x; e_x_, age specific expectation of life in years.

## Discussion

The present study detected a significant ontogenetic variation of SSD in overall body size and in proportional morphological dimensions in *P. przewalskii*, which defined the critical periods in which male and female sizes diverge. The SSD in overall body size was not significant until after sexual maturation but differences between the two sexes in the other proportional morphological traits were significant just before sexual maturation.

Like most other agamids [1], our lizards exhibited male-biased SSD in SVL, TL, HW and LL among adults. Male-biased SSD is usually considered in terms of ultimate mechanisms of sexual selection and sexual niche segregation [1]. The niche divergence hypothesis, which mainly operates through reduced competition between male and female lizards, was popular for reptiles, especially for gap-limited species. However, this is not applicable to this species because no sexual difference was found in prey composition or size (Zhao and Liu, unpub. data). Sexual selection is generally thought to favor larger male lizards, mainly through increased success in behavioral interactions [2], [20]. Moreover, head size generally relates to bite force [21], [22]. Increased head size may be related to the increased bite force necessary to win male-male contests and also may be useful in copulatory interactions. Additionally, limb and tail lengths in lizards are often correlated with speed [23] so that male lizards with long limbs and tails would have advantages for territory defense and maintenance [24], [25]. In on our field research, male-male combat, male tail-curling displays, and male-female chasing in *P. przewalskii* were common during the reproductive season (Zhao and Liu, unpub. data). In conclusion, male-biased SSD in *P. przewalskii* may be ultimately caused by sexual selection.

For species that continue to grow after maturation, sex-specific mortality resulting from the greater cost of reproduction in female than male species always leads to unequal age distribution [8], [26], which in turn gives rise to male-biased SSD in overall body size. This alternative proximate explanation for male-biased SSD does not seem to apply to *P. przewalskii*. Our survivorship and life-table analyses both confirmed that male lizards suffered higher mortality, especially during the first 3 years of life. Male-biased mortality in this population may result from male-male aggression. First, male-male combat may force smaller male lizards to disperse [27], appearing as increased mortality in young male lizards. Second, displaying and fighting are both energetically expensive and potentially increase exposure to predators [28]. Our field studies confirmed that male lizards defended territory by tail-curling displays during the breeding season and were more vulnerable than female lizards (Zhao and Liu, unpub. data).

Sexual difference in growth pattern is another possible proximate mechanism for the male-biased SSD [7], [9], [29]. In *P. przewalskii*, male-biased SSD in SVL was fully expressed at the age of 11 months (June after hatching) and the difference in age-specific linear growth rates between two sexes was also significant at age 10–11 months. This synchrony suggests that growth difference was involved in the development of SSD. The same synchronies were also found in TL and LL. That is, the sexual difference of the growth rate was the proximate cause of male-biased SSD in *P. przewalskii*. The SSD of HW was fully developed just after hibernation, however the growth of hibernation was not considered. So we failed to find the synchrony between linear growth rate and the development of SSD in HW. Interestingly, we found that the development of SSD in TL, HW, and LL occurred earlier than it did for SVL. Many researchers have considered that performance is more important than body size in male-male competition [29]–[32]. However, our findings suggested that male lizards of *P. przewalskii* experienced sexual selection and also confirmed that TL, HW, and LL were important in competition.

Why do male lizards grow faster than female lizards? Several alternatives are possible for this sexual differentiation. Obviously, individual growth rates are related to food consumption [4], [9]. Different habitats occupied by male and female lizards are typically associated with differences in prey availability and could also be a cause of SSD [33]. However, no habitat difference was observed in this population [16]. Changes in the feeding rate of one sex in the absence of any difference in resource availability could also give rise to differential growth rates. To support follicular development, vitellogenesis and ovulation during breeding season, female lizards must maintain their body temperature within a relatively narrow range. Thermoregulatory behavior takes up much activity time and, therefore, may reduce the time available for feeding. Although we did not investigate the time budget of *P. przewalskii*, this mechanism seems unlikely. The fecundity of female *P. przewalskii* is correlated with maternal size [15], so the feeding cost is expected to increase as the lizards grow. Therefore, we predict that sexual difference in growth would be greater among the elder age classes. However, this sexual difference is only observed before maturity in *P. przewalskii*.

Trade-offs between growth and reproduction, one of the central assumptions of life history theory, is a further alternative mechanism of SSD. Experiments employing surgical treatments (e.g., ovariectomy) have confirmed that the energetic cost of reproduction reduces the growth of female lizards to some extent [29], [34]. However, the energetic and time costs of reproductive behavior could slow the growth of male lizards [35]. This may explain why we observed no sexual difference in growth after sexual maturation in *P. przewalskii*. The observation that yearling male lizards grow more quickly than female lizards may be attributed to their different reproductive states. Under the stress of male-male competition few young male lizards have an opportunity for reproduction before they have established their own territory. In contrast, female lizards can participate in reproduction as soon as they reach sexual maturity [12], [17].

It is possible that the process of sexual divergence may reflect sexual differences in the profile of growth-regulating hormones [6]. The sex steroid testosterone may act as a bipotential growth regulator by stimulating growth in species with males-larger SSD and inhibiting growth in species with female-larger SSD [4], [6], [36]. In natural populations of *Sceloporus jarrovii*, castration slowed the growth of yearling male lizards, while treatment of castrated males with exogenous testosterone reduced the effect of castration [4]. It is worth noting that this natural sexual difference in growth can be eliminated by certain environmental conditions [4], [37]. Further research is needed to clarify the relative importance of genetic versus environmental control of SSD in natural populations.

In summary, using mark-recapture methods, the proximate cause of male-biased SSD in *P. przewalskii* and the critical periods in which male and female sizes diverge, were determined. Male lizards exhibited higher mortality than female lizards and grew faster immediately prior to the establishment of SSD. Two predictions of the sexual selection hypothesis were confirmed in our study: mature male lizards experience high mortality associated with territoriality and male lizards reach a higher asymptotic size than female lizards. Thus, we suggest that the male-biased SSD in *P. przewalskii* was ultimately caused by sexual selection and proximately caused by sexual difference in growth. We also found that performance-related characteristics such as TL, HW, and LL diverged earlier than SVL, which indicated that they were exposed to stronger sexual selection than was SVL. Differential energy allocation induced by difference in reproductive status may be the underlying physiological mechanism of the sexual difference in growth.
